# Adiponectin promoter polymorphisms are predictors of lipid profile improvement after bariatric surgery

**DOI:** 10.1590/1678-4685-GMB-2016-0241

**Published:** 2017-10-23

**Authors:** Aline Simas Gasparotto, Diego Olschowsky Borges, Marília Remuzzi Zandoná, Mauricio Jacques Ramos, Nelson Guardiola Meihnardt, Vanessa S. Mattevi

**Affiliations:** 1 Programa de Pós-Graduação em Ciências da Saúde, Universidade Federal de Ciências da Saúde de Porto Alegre, Porto Alegre, RS, Brazil; 2 Centro de Atenção ao Obeso Classe III, Hospital Nossa Senhora da Conceição, Grupo Hospitalar Conceição, Porto Alegre, RS, Brazil

**Keywords:** Adiponectin, polymorphism, obesity, lipid profile, bariatric surgery

## Abstract

Our aim was to investigate if single nucleotide polymorphisms (SNPs) located in the 5′ regions of leptin (*LEP*, -2548 G > A, rs7799039), resistin (*RETN*, -420 C > G, rs1862513) and adiponectin (*ADIPOQ*, -11391 G > A, rs17300539 and -11377 C > G, rs266729) genes were related to changes in body mass index (BMI) and metabolic variables after bariatric surgery in 60 extremely obese individuals. At baseline, *ADIPOQ* -11391 A-allele carriers showed higher plasma adiponectin and lower total cholesterol levels when compared to G/G homozygotes. Approximately 32 months post-surgery, a mean reduction of 35% in BMI and an important improvement in metabolic profiles were observed. In addition, for the *ADIPOQ* -11377 polymorphism, a higher decrease in lipid profile was associated to the C/C genotype. Moreover, individuals bearing the A-C haplotype for the two *ADIPOQ* SNPs were more prone to show a reduction in low-density lipoprotein levels after bariatric surgery (-43.0% A-C carriers vs. -18.1% G-G carriers, p = 0.019). We did not find any association of leptin and resistin SNPs with the clinical parameters analyzed. In summary, our results indicate that the A-C haplotype is a predictor of better lipid profile post-surgery and the studied SNPs in *ADIPOQ* gene are associated to changes in metabolic variables in obese individuals.

## Introduction

Obesity is a health problem that affects not only the welfare of individuals worldwide, but also the economy, representing a heavy burden to public health systems (Krzysztoszek *et al.*, 2015). To loose and maintain weight loss is not an easy goal for most people. Efficacious and safe pharmacological treatments are still lacking and lifestyle modifications are currently the first choice treatment to the excess of body weight. Surgical interventions are being highly used to treat patients with morbid obesity (body mass index, BMI, equal or over 40 kg/m^2^). However, there is a high inter-subject variability among surgical outcomes (Sevilla and Hubal, 2014).

Genetic factors have been demonstrated to explain almost 70% of BMI variability (Visscher *et al.*, 2012; Zaitlen *et al.*, 2013). Due to these high estimates of body weight heritability and to inter-individual differences observed in response to bariatric surgery, the role of individual genetic background on outcomes of this intervention becomes an important gap in the present knowledge.

Since 1948, proteins secreted by adipose tissue, *i.e.* adipokines, have been related to energy expenditure regulation through both central and endocrine actions (Wertheimer and Shapiro, 1948). Moreover, excess of adipose tissue in obesity has been shown to disturb adipokine signaling and to be linked to insulin resistance, hyperglycemia, increased risk of cardiovascular diseases and dyslipidemia (Antuna-Puente *et al.*, 2008; Leal and Mafra, 2013). Three adipokines, namely leptin, resistin, and adiponectin, known to play important roles in the modulation of the metabolic adverse effects associated with the excess of adipose tissue, have been chosen as the focus of the present study. Leptin acts inhibiting appetite and food intake and stimulating energy expenditure. However, circulating leptin levels produced by adipose tissue are increased in obese subjects, probably due to the existence of leptin resistance. Resistin is also an adipocyte-specific secreted adipokine with conflicting reports of its potency in metabolic diseases in humans. However, several studies have consistently reported a close relationship between resistin levels and obesity, insulin resistance, or type 2 diabetes. On the other hand, adiponectin levels are low in obese subjects, and this adipokine produces insulin-sensitizing effects (reviewed in Jung and Choi, 2014). Therefore, it has been proposed that genetic variation influencing adipokine action can also alter many physiological and pathological mechanisms (Breitfeld *et al.*, 2012).

Variations in regulatory regions of genes encoding adipokines have been associated with obesity-related phenotypes by our group and others (Mammes *et al.*, 2000; Mattevi *et al.*, 2002, 2004; Norata *et al.*, 2007; Ben Ali *et al.*, 2009). These findings strengthen the connection between adipokines and obesity. However, the role of these variants in longitudinal studies and in response to bariatric surgery has been far less investigated. Therefore, our aim was to investigate if single nucleotide polymorphisms (SNPs) located in the 5’ regions of leptin, resistin and adiponectin genes are related to a different profile of weight loss and/or changes in metabolic variables evaluated before and after gastric reduction surgery in obese individuals.

## Materials and Methods

### Subjects

Sixty obese subjects undergoing elective gastric bypass abdominal surgery [Roux-en Y gastroenterostomy, which has been considered the gold standard for surgical treatment of obesity in the United States (Barnett, 2013)] were recruited at the Obese Class III outpatient clinic at a government-supported hospital in Rio Grande do Sul, Brazil. Inclusion criteria were body mass index [BMI, calculated as (weight in kg) / (height in m)^2^] equal or over 40 kg/m^2^ or BMI equal or over 35 kg/m^2^ with associated comorbidities (type 2 diabetes, sleep apnea, hypertension, dyslipidemia, cardiovascular diseases, or osteoarthritis). Individuals were excluded in presence of serious hepatic disease, cancer, coagulation disorders or stomach diseases.

In order to characterize the sample, information about birth, ethnicity, gender, physical activity, smoking, use of oral contraceptives, menopausal status, weight, height, presence of type 2 diabetes and hypertension were obtained from medical files recorded at the time of the surgery. In addition, as part of the patient’s routine care, laboratory variables information (fasting total cholesterol, low-density lipoprotein, high-density lipoprotein, glucose, triglycerides and glycosylated hemoglobin) were collected in two different moments: a) pre-surgery (baseline); and b) after 32 ± 7 months post-surgery. Serum insulin and reactive C protein levels were available for only a few individuals, so these variables were not further analyzed.

Plasma levels of leptin, resistin and adiponectin were measured in pre-surgery fasting blood samples using the Human Leptin, Resistin, and Adiponectin Elisa Kits, respectively (EMD Millipore Corporation, Missouri, USA). Plasma samples for these measurements were available for only 43 individuals.

### SNPs genotyping

Fasting whole blood samples were collected during surgery and DNA was extracted using a standard salting out technique (Lahiri and Nurnberger Jr., 1991). We conducted polymerase chain reactions (PCR) targeting regions containing polymorphisms located in the genes encoding leptin (*LEP* -2548G > A; rs7799039, data available for 53 individuals due to unsuccessful genotyping of some samples) and resistin (*RETN* -420C > G; rs1862513, available for 50 individuals) using primers and reaction conditions previously described (Mammes *et al.*, 2000; Engert *et al.*, 2002). Digestion of PCR products with the enzymes *Hha*I for *LEP*-2548 G > A and *Bpi*I for *RETN* -420C > G was conducted afterwards. Restriction fragment length analysis was performed in agarose gels (2.5%) containing ethidium bromide to determine genotypes. Two variants in the promoter region of the adiponectin (*ADIPOQ*) gene (-11377 C > G; rs266729, obtained for 57 individuals and -11391 G > A; rs17300539, obtained for 56 individuals) were genotyped in a StepOnePlus real-time PCR System (Life Technologies^®^, California, USA), using hydrolysis probes for allele discrimination. Negative and positive controls were included in all analyses. Five percent of all samples were repeated for genotype confirmation. Selection of SNPs was based in previous results obtained by our group regarding their associations with anthropometric or metabolic phenotypes in the Brazilian population (Mattevi *et al.*, 2002, 2004; Trinca *et al.*, 2010).

### Statistical analysis

Variables used for sample characterization are expressed as mean ± standard deviation or frequency (%). Asymmetrically distributed continuous variables are presented as medians [interquartile range]. Allele frequencies were calculated and agreement of genotype frequencies with Hardy-Weinberg expectations was tested through a goodness-of-fit chi-squared test. Pairwise linkage disequilibrium coefficients (D’ standardized linkage disequilibrium, and r^2^, squared correlation coefficient) were estimated using the Haploview software (Barrett *et al.*, 2005).

Mean biochemical and anthropometric parameters at baseline and % change after surgery were compared among the different genotypes using Kruskal-Wallis and Mann-Whitney-U tests. Prior to analyses, the changes in biochemical and anthropometric variables were adjusted by linear regressions for the time interval between data collections. Age, menopausal status and gender did not show significant correlation with biochemical and anthropometric measurements in previous univariate analyses; therefore, they were not included in adjustments. All tests were two-tailed, and the significance level was pre-defined at *p* < 0.05. Data presented herein are limited to exploratory analyses and, therefore, multiple tests correction has not been performed. All statistical analyses were conducted using IBM SPSS Statistics version 20.0.0 (SPSS Inc., Chicago, USA).

### Ethical considerations

Informed consent was obtained from all individual participants included in the study. All procedures performed in this study were in accordance with the ethical standards of the institutional research committees (protocol numbers 11-105, UFCSPA, and 481/11, Grupo Hospitalar Conceição).

## Results

Selected clinical characteristics of the 60 individuals from this study are presented in Table 1. The studied sample was almost completely constituted by women (91.7%) and euro-descendants (95.0%), with a mean age of 42.3 ± 8.9 years. The majority of patients did not smoke (93.3%) and 83.6% of women did not make use of oral contraceptives. Six of the enrolled women were postmenopausal. Other clinical variables at baseline are described in Table 1.

**Table 1:** Baseline clinical characteristics of the 60 morbidly obese enrolled subjects.

Characteristic	*
Age (years)	42.3 ± 8.9
Women	55 (91.7%)
Euro-descendants	57 (95.0%)
Smoking	4 (6.7%)
Sedentary	50 (83.3%)
Type 2 diabetes	32 (53.3%)
Hypertension	44 (73.3%)
Dyslipidemia	36 (60.0%)
Metabolic syndrome	40 (66.7%)
BMI (kg/m^2^)	50.7 ± 7.8
Abdominal subcutaneous fat thickness (cm)	6.6 ± 1.8
Leptin (ng/mL)^a^	37.3 [24.7-47.3]
Adiponectin (ug/mL)^a^	12.5 [10.2-19.5]
Resistin (ng/mL)^a^	0.59 [0.43-0.80]

a Adipokine levels available for 43 individuals;

* Data are shown as mean ± SD, median [interquartile range] or absolute n (%). BMI, body mass index.

All genotype frequencies were in agreement with those expected under Hardy-Weinberg equilibrium. Minor allele frequency for *LEP* -2548 G > A was 0.39 (A), for *RETN* -420C > G was 0.31(G), for *ADIPOQ* -11391 G > A was 0.11 (A), and for *ADIPOQ* -11377 C > G was 0.31 (G). Three haplotypes resulting from the combination of the two SNPs in the *ADIPOQ* gene were observed, G-C, G-G and A-C, with the following frequencies: 0.58, 0.31 and 0.11, respectively. Linkage disequilibrium coefficients were also calculated for these two SNPs. D’ and r-squared values were 0.208 and 0.002, respectively.

The relationship between each one of the SNPs located in *LEP*, *RETN* and *ADIPOQ* coding genes and circulating concentrations of their respective proteins was investigated through comparison of median adipokine levels among genotypes (Table 2). *ADIPOQ* -11391 A-allele carriers showed higher plasma adiponectin levels when compared to homozygotes for the G allele (19.5 versus 12.4 ug/ml, p = 0.045, respectively). Leptin and resistin plasma levels were not different among genotypes for SNPs located in their respective encoding genes.

**Table 2:** Comparison of adipokines (leptin, resistin and adiponectin) plasma levels among their respective encoding-gene polymorphisms genotypes before surgery.

Polymorphism	Genotype (n)	Adipokine*	Interquartile range
		Leptin (ng/mL)	
*LEP* -2548 G > A (rs7799039)	G/G (24)	36.4	24.8 – 47.8
	A/G (17)	37.3	22.0 – 50.1
	A/A (12)	39.4	32.6 – 42.3
	*p*	0.988^a^	
		Resistin (ng/mL)	
*RETN* -420 C > G (rs1862513)	C/C (22)	0.64	0.44 – 0.92
	G/G+C/G (28)	0.59	0.42 – 0.76
	*p*	0.422^b^	
		Adiponectin (μg/mL)	
*ADIPOQ* -11391 G > A (rs17300539)	G/G (45)	12.4	1.2 – 17.7
	A/A+A/G (11)	19.5	11.9 – 30.4
	*p*	0.045^b^	
*ADIPOQ* -11377 C > G (rs266729)	C/C (26)	13.6	10.9 – 18.9
	G/G+C/G (31)	12.5	10.2 – 25.4
	*p*	0.921^b^	

* Data are expressed as median and interquartile range;

a Kruskal-Wallis test;

b Mann-Whitney U test; n < 60 due to unsuccessful genotyping of some samples.

In addition, we compared BMI and other biochemical parameters [triglycerides, total cholesterol, low density lipoprotein (LDL) and high density lipoprotein (HDL) cholesterol, glycosylated hemoglobin and glucose levels] among genotypes before surgery. *ADIPOQ* -11391 G/G homozygotes showed higher total cholesterol levels than A-allele carriers (212.1 vs. 182.9 mg/dL, *p*= 0.019). Other parameters were not different between these and other genotypes (data not shown).

The second data collection for blood analysis was performed after 32 ± 7 months post-surgery. Only one patient was lost after follow-up. As shown in Table 3, mean BMI reduction was 35.0% after this period. All metabolic parameters evaluated presented lower values after the surgical procedure, with exception of HDL-cholesterol, which was higher than before surgery, as expected.

**Table 3:** Evolution of anthropometric and metabolic parameters following bariatric surgery.

	Baseline	After surgery	Variation	Variation (%)	*p*	n
BMI (kg/m^2^)	50.3 ± 1.0	32.8 ± 0.7	-17.5 ± 0.8	-35.0	< 0.001 a	59
Total cholesterol (mg/dl)	202.7 ± 5.1	178.0 ± 3.7	-24.7 ± 5.0	-10.7	< 0.001 a	57
LDL cholesterol(mg/dl)	125.2 ± 4.6	93.5 ± 3.3	-31.8 ± 4.2	-22.6	< 0.001 a	56
HDL cholesterol (mg/dl)	48.8 ± 1.3	66.9 ± 1.9	18.2 ± 1.7	41.2	< 0.001 a	57
Triglycerides (mg/dl)	142.6 ± 8.0	87.5 ± 3.5	-55.1 ± 1.3	-40.3	< 0.001 b	56
Glucose (mg/dl)	129.1 ± 7.7	91.8 ± 2.7	-37.3 ± 1.2	-21.8	< 0.001 b	58
Glycosylated hemoglobin (%)	6.7 ± 0.3	5.6 ± 0.8	-1.1 ± 0.2	-13.4	< 0.001 b	53

Data are presented as means ± SEM.

a Paired samples t-test;

b Wilcoxon test. Second data collection performed 35 ± 6 months (for BMI) and 32 ± 7 months (for the other parameters) after surgery; n < 60 due to unsuccessful genotyping of some samples. BMI, body mass index; LDL, low-density lipoprotein; HDL, high-density lipoprotein.

**Figure 1:**
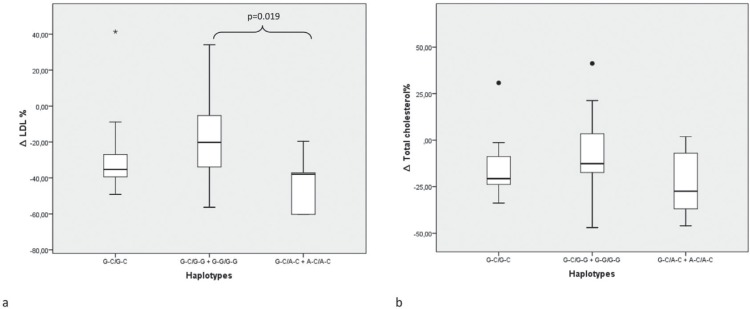
Comparison of percent variation in total cholesterol and low-density lipoprotein levels following bariatric surgery among *ADIPOQ* -11391 G > A (rs17300539) and -11377 C > G (rs266729) haplotype combinations. (A) Comparison of median %-variation in LDL levels among *ADIPOQ* haplotype combinations. Kruskal-Wallis test among the three haplotype combinations, p = 0.029; Mann-Whitney test used to show difference between G-G carriers and A-C carriers medians, *p*= 0.019. *Outlier individual value of ΔLDL%. ΔLDL%, percentual variation in low-density lipoprotein levels. (B) Comparison of median % variation in total cholesterol levels among *ADIPOQ* haplotype combinations. Kruskal-Wallis test among the three haplotype combinations, p = 0.178. • Outlier individual values of Δ total cholesterol %. Δ, variation. Data are shown as median and interquartile range adjusted for the time interval between data collections.

The possible relationship of the gene polymorphisms investigated with response to bariatric surgery was evaluated through comparison of mean BMI variation and changes in metabolic variables (lipid and glucose serum levels) between genotypes of the studied SNPs. *ADIPOQ* -11377 C/C individuals have shown higher reduction of LDL cholesterol, total cholesterol and triglycerides when compared to G-allele carriers (Table 4). When this analysis was conducted among *ADIPOQ* -11391 and -11377 haplotype combinations, a higher LDL cholesterol reduction was associated to A-C haplotype carriers when compared to carriers of the G-G haplotype (Figure 1, -43.0 vs. -18.1%, *p*= 0.019). For other genetic variants analyzed no statistical significant differences were found (data not shown).

**Table 4:** Percent change in circulating lipids following bariatric surgery according to *ADIPOQ* -11377 C > G (rs266729) genotypes.

	C/C	G/G +C/G	*p*
Δ LDL %	-37.1 [-40.2 – -20.0]	-18.3 [-32.3 – -4.9]	0.005^a^
n	23	30	
Δ Cholesterol %	-20.7 [-27.5 – -7.0]	-8.8 [-15.6 – 3.4]	0.038^a^
n	23	31	
Δ Triglycerides %	-43.1 ± 22.0	-31.1 ± 19.5	0.042^b^
n	22	31	

All variables have been adjusted for time interval between data collection. Data are expressed as mean ± standard deviation or median and [interquartile range].

a Mann-Whitney U test;

b t-Test for independent samples; n < 60 due to unsuccessful genotyping of some samples. Δ, variation; LDL, low-density lipoprotein.

## Discussion

Bariatric surgery has been shown to be an effective treatment for extreme obesity. Morbid obesity is usually associated with development of dyslipidemia, insulin resistance and other metabolic disturbances (Gutierrez *et al.*, 2009; Farb *et al.*, 2011). In our sample, mean BMI reduction was 35.0% after surgical intervention. Our data also show an important improvement in lipid and glycemic profiles after bariatric surgery. However, the relationship between genetic background and bariatric surgery outcomes remains to be elucidated. In the present study, we longitudinally examined the possible role of polymorphisms located in three adipokine encoding genes in the modulation of surgical weight loss and lipid and glucose-related metabolic parameters.

Adiponectin was identified in the 1990s decade, being the most abundant transcript produced by adipose tissue (Maeda *et al.*, 1996). Plasma adiponectin levels are known to be negatively correlated with body fat mass. It is also well-known that adiponectin has insulin-sensitizing effects. This protein also induces activation of lipoprotein lipase enzyme, thereby enhancing very-low density lipoprotein clearance and thus decreasing plasma triglyceride levels (Berneis and Krauss, 2002), therefore acting as an anti-atherogenic adipokine. In addition, adiponectin has important anti-inflammatory properties. Although not fully established, this adipokine could act by reducing anti-inflammatory molecules expression and by inhibiting macrophage transformation to foam cells, for example (Ouchi and Walsh, 2007).

Although all individuals analyzed herein were extremely obese in a stable weight situation before surgery, we observed that A-allele carriers for the *ADIPOQ* -11391G > A SNP showed higher adiponectin plasma levels than G-allele homozygotes. Similar results were previously reported in a study performed in obese subjects before bariatric surgery in France (Poitou *et al.*, 2005) and also by our group in a sample of HIV-infected patients on highly-active antiretroviral therapy, where A-allele carriers showed higher adiponectin plasma levels than other genotypes (Trinca *et al.*, 2010). Furthermore, we observed that A-allele carriers had lower baseline total cholesterol levels than G/G homozygotes for this SNP. Even though similar results related to the lipid profile were not found in the literature, the *ADIPOQ* -11391 A-allele has been already associated with lower risk of type 2 diabetes in obese adults (Vasseur *et al.*, 2005; Goyenechea *et al.*, 2009). For this reason, we believe that the presence of this allele could imply in a metabolic advantage for obese individuals but this needs to be further explored in future studies.

For the second *ADIPOQ* polymorphism analyzed, -11377 C > G, C-allele homozygotes presented higher reduction in LDL cholesterol, total cholesterol and triglycerides than carriers of the variant G-allele. Higher levels of total and LDL cholesterol were associated to the G/G genotype in a previous study by our group performed in 3-4 years-old children from Brazil (Zandona *et al.*, 2013). Although there were differences in enrolled individuals, both results suggest the C/C genotype as a predictor of a better response in circulating lipids after surgery, since the higher decreases were reported in this group.

Both studied SNPs are located in the promoter region of the *ADIPOQ* gene and have already been associated with adiponectin levels (Bouatia-Naji *et al.*, 2006). However, previous characterization of the *ADIPOQ* gene promoter did not identify binding sites for transcription factors at or around both SNPs (Schaffler *et al.*, 1998). Although linkage disequilibrium coefficients between the two variants studied in the *ADIPOQ* gene were quite low in the present sample, we (Trinca *et al.*, 2010) and others (Poitou *et al.*, 2005) have found in previous studies a significant linkage disequilibrium between these two SNPs. Thus, we believe that the small D’ and r^2^ estimates found herein are most likely due to sample size, because we had a low number of carriers of the -11391 A allele (only 11). Evidence presented herein allow us to speculate that these SNPs may be in linkage disequilibrium with an unknown functional site in this region, which would explain the consistent associations of the A-C haplotype with adiponectin levels found in different studies, as also suggested by Kyriakou *et al.* (2008).

Associations of *LEP* -2548 G > A and *RETN* -420C > G SNPs with the plasma levels and other metabolic parameters mentioned above were not found. The same result was reported by our group for the *LEP* gene SNP in human immunodeficiency virus (HIV)-infected individuals (Trinca *et al.*, 2010), by Ortega *et al.* (2014) in prepubertal children and adolescents, and by Menzaghi *et al.* (2006) in non-diabetic Caucasians for the *RETN* variant. The results suggest that the variants located at leptin and resistin genes are not associated with the parameters determined in this study.

One limitation of our study is the high proportion of women in our sample, limiting the extrapolation of our results to the male gender. Another weak point is the lack of post-surgical adipokine measurements, which would have enriched our results. We are also aware that only a few polymorphisms in the candidate genes were analyzed in the present study, so we cannot rule out the role of other variants in these genes on the modulation of the outcomes of bariatric surgery.

In summary, based on the data presented herein we suggest that morbid obese individuals bearing the A-C haplotype for the *ADIPOQ* -11391 and -11377 SNPs are prone to show a higher reduction in circulating lipid levels after bariatric surgery than other subjects, therefore having more benefits from this intervention. Future studies should focus on the role of the adiponectin-signaling pathway over weight loss interventions and improvement in circulating lipids profile to validate these results and to introduce genetic analyses in evaluations before clinical practice decisions.
